# Feeding intolerance alters the gut microbiota of preterm infants

**DOI:** 10.1371/journal.pone.0210609

**Published:** 2019-01-22

**Authors:** Zhenya Yuan, Junmei Yan, Hongyu Wen, Xiaoyi Deng, Xianbin Li, Siting Su

**Affiliations:** 1 School of Life Science, Jiangsu Normal University, Xuzhou, China; 2 Xuzhou Maternity and Child Health Care Hospital, Xuzhou, China; University of Illinois, UNITED STATES

## Abstract

Feeding intolerance (FI) is a common disease in preterm infants, often causing a delay in individual development. Gut microbiota play an important role in nutrient absorption and metabolism of preterm infants. To date, few studies have focused on the community composition of gut microbiota of preterm infants with feeding intolerance. In this study, we collected fecal samples from 41 preterm infants diagnosed with feeding intolerance and 29 preterm infants without feeding intolerance, at three specific times during the development and prevalence of feeding intolerance (after birth, when feeding intolerance was diagnosed, after feeding intolerance was gone), from different hospitals for 16S rRNA gene sequencing. The gut microbiota community composition of preterm infants diagnosed with feeding intolerance was significantly different from that of preterm infants without feeding intolerance. At the time when feeding intolerance was diagnosed, the relative abundance of *Klebsiella* in preterm infants with feeding intolerance increased significantly, and was significantly higher than that of the preterm infants without feeding intolerance. After feeding intolerance was cured, the relative abundance of *Klebsiella* significantly decreased in the infants diagnosed with feeding intolerance, while the relative abundance of *Klebsiella* in preterm infants without feeding intolerance was not significantly altered during the development and prevalence of feeding intolerance. Furthermore, we verified that *Klebsiella* was effective in the diagnosis of feeding intolerance (AUC = 1) in preterm infants, suggesting that *Klebsiella* is a potential diagnostic biomarker for feeding intolerance.

## Introduction

Early feeding plays an important role in individual development of preterm infants after birth [1]. Feeding intolerance is a common disease caused by an immature gastrointestinal tract or intestinal function disorder in preterm infants [2]. Although a universal definition of feeding intolerance is still lacking, preterm infants with a gastric residual volume of more than 50% of the previous feeding volume, gastric regurgitation, abdominal distension and/or emesis, indicating an inability to digest enteral feeding are diagnosed with feeding intolerance [3,4]. Feeding intolerance often leads to sub-optimal nutrition, postnatal growth restriction and several adverse effects on normal development, which are serious threats to the survival of preterm infants [5]. Moreover, the retardation of progress in achieving enteral feeding caused by feeding intolerance constantly leads to prolonged parenteral feeding with an increased risk of nosocomial infections and delays in hospital discharge.

With the development of next-generation sequencing technologies, the microbiota of the human gut has been deemed as “another” genome [6,7]. Gut microbial colonization has been proven to be potentially initiated prenatally [8]. In the perinatal stage, the community composition of gut microbiota can be altered by factors such as the route of delivery, antibiotic exposure, gestational age at birth, route of feeding and gender. Therefore, colonization patterns and evolutionary processes of the gut microbiota are diverse in preterm infants [9,10]. The community composition of the gut microbiota is quite unstable and of great individual variation in preterm infants that impacts individual metabolic, immunologic and nutritional processes [11,12]. A harmonious and symbiotic relationship between gut microbiota and the host is conducive in preventing pathogen colonization in order to promote digestion, to improve intestinal barrier function, to modulate the immunoreaction, to facilitate the metabolic and signal transduction pathways of gut-brain cross-talk and to assist in maintaining homeostasis [13,14]. Additionally, probiotic groups of the human gut have been confirmed to be associated with dramatic decreases in feeding intolerance morbidity and quicker attainment of full enteral feeding [15,16].

Human diseases are most often associated with the variations in the gut microbiota, and the significance of gut microbiota has drawn the public attentions. Gut microbiota are also inchoate diagnostic markers of many diseases and are pharmacological targets for drugs [17–20]. The clinical symptoms of feeding intolerance in preterm infants are quite similar to the early presentations of necrotizing enterocolitis (NEC), which is a severe disease with a leading mortality of preterm infants [4,21]. Although the detailed pathogenic mechanism of NEC is still unclear, dysbacteriosis of the gut microbiota of preterm infants after birth is acknowledged as a potential pathogenesis of NEC [21]. Previous studies have described the specific community composition and the metabolic characteristics of gut microbiota of preterm infants with NEC, which are important in searching for microbial or molecular diagnostic markers of NEC [22–24]. However, the variation in gut microbial community composition which is vital in developing potential diagnostic markers of feeding intolerance in preterm infants is still unknown.

To date, most studies on feeding intolerance in preterm infants have focused on the clinical manifestations of preterm infants after treatment and the efficacy of probiotics in curing feeding intolerance, while there are a few clinical trials that have studied the characteristics and variation in gut microbial community composition of preterm infants with feeding intolerance. Diagnostic markers of feeding intolerance are still undefined as well. Therefore, studies are needed to explore the relevance of the gut microbiota in feeding intolerance. A case-control study was conducted to detect the difference between the gut microbial community composition of 41 preterm infants diagnosed with feeding intolerance and 29 premature infants without feeding intolerance at different stages in the development and prevalence of feeding intolerance, using high-throughput sequencing methods on 16S rRNA genes of the samples. In our study, the community composition of the gut microbiota of preterm infants with feeding intolerance was greatly altered when feeding intolerance was diagnosed. Furthermore, when feeding intolerance was diagnosed, the comparison of gut microbiota between the infants with feeding intolerance and the infants without feeding intolerance revealed huge differences. We also found that certain microbes that were significantly different in samples were potential diagnostic markers of feeding intolerance.

## Subjects and methods

### The diagnosis criteria of feeding intolerance

Preterm infants with presentations of a gastric residual volume of more than 50% of the previous feeding volume and abdominal distension were diagnosed as feeding intolerant at Xuzhou Maternity and Child Health Care Hospital in Xuzhou, China and at Nanjing Maternity and Child Health Care Hospital in Nanjing, China. The infants with feeding intolerance often had accompanying presentations of gastric regurgitation and/or emesis. We defined that feeding intolerance was cured when the infants had neither of the presentations of a gastric volume more than 50% of the previous feeding volume, abdominal distension, gastric regurgitation or emesis.

### Exclusion and inclusion criteria

This case-control study was conducted at Xuzhou Maternity and Child Health Care Hospital in Xuzhou, China and at Nanjing Maternity and Child Health Care Hospital in Nanjing, China. 43 preterm infants were admitted from January to June 2017 from Xuzhou Maternity and Child Health Care Hospital, and 27 preterm infants were admitted from January to July 2018 from Nanjing Maternity and Child Health Care Hospital, with a gestational age of less than 37 weeks. 26 preterm infants diagnosed with feeding intolerance and 17 preterm infants without feeding intolerance were admitted at Xuzhou Maternity and Child Health Care Hospital, while 15 preterm infants diagnosed with feeding intolerance and 12 preterm infants without feeding intolerance were admitted at Nanjing Maternity and Child Health Care Hospital. All 41 infants diagnosed with feeding intolerance were assigned to the feeding intolerance group (FIG) and the 29 infants without feeding intolerance were assigned to the control group (CG). The groups of preterm infants from Xuzhou Maternity and Child Health Care Hospital were named as FIG_X and CG_X, while the groups of preterm infants from Nanjing Maternity and Child Health Care Hospital were named as FIG_N and CG_N.

The inclusion criteria for this study was as follows:

(1) The gestational age of the infant had to be less than 37 weeks. (2) The preterm infant was assigned to the FIG group if it was diagnosed with feeding intolerance and its condition improved to attain full enteral feeding through clinical treatments during hospitalization. (3) The preterm infants assigned to the CG group had to have never been diagnosed with feeding intolerance during hospitalization. (4) The preterm infants assigned to FIG and CG groups had to have never left the incubators before being transferred to another hospital or being discharged from the Newborn Medical Center of the Maternity and Child Health Care Hospital.

The exclusion criteria for this study was as follows:

(1) Infants who suffered from systemic inflammatory reaction, Crohn’s disease, IBD (inflammatory bowel disease), congenital gastrointestinal anomalies, necrotizing enterocolitis (NEC), pneumonia or severe diarrhoea during hospitalization. (2) Infants who had severe asphyxia, coagulation disorders or those who had received an abdominal surgery operation during hospitalization. (3) Infants whose mothers had received antenatal antibiotic treatment. (4) Infants whose presentations of feeding intolerance reappeared within 7 days after feeding intolerance was cured.

Parents were well informed about this study and written informed consent was obtained from the parents of each infant regarding participation. Patient anonymity was preserved in this study. We declare that only fecal samples were collected from the infants. We ensured that there was no extra force or drug given to promote defecation of infants and the collection caused no pain or damage to the infants. This study caused no interference with the clinical judgement and decision of the physician. All procedures of this study were approved by the Clinical Research Ethics Committee of the Xuzhou Maternity and Child Health Care Hospital (Approval Number 2017FL016) and the Clinical Research Ethics Committee of the Nanjing Maternity and Child Health Care Hospital (Approval Number FL(2016)73). The WHO clinical trial registration number of this study is ChiCTR-ROC-17012180.

### Fecal sampling

**Fig 1:**
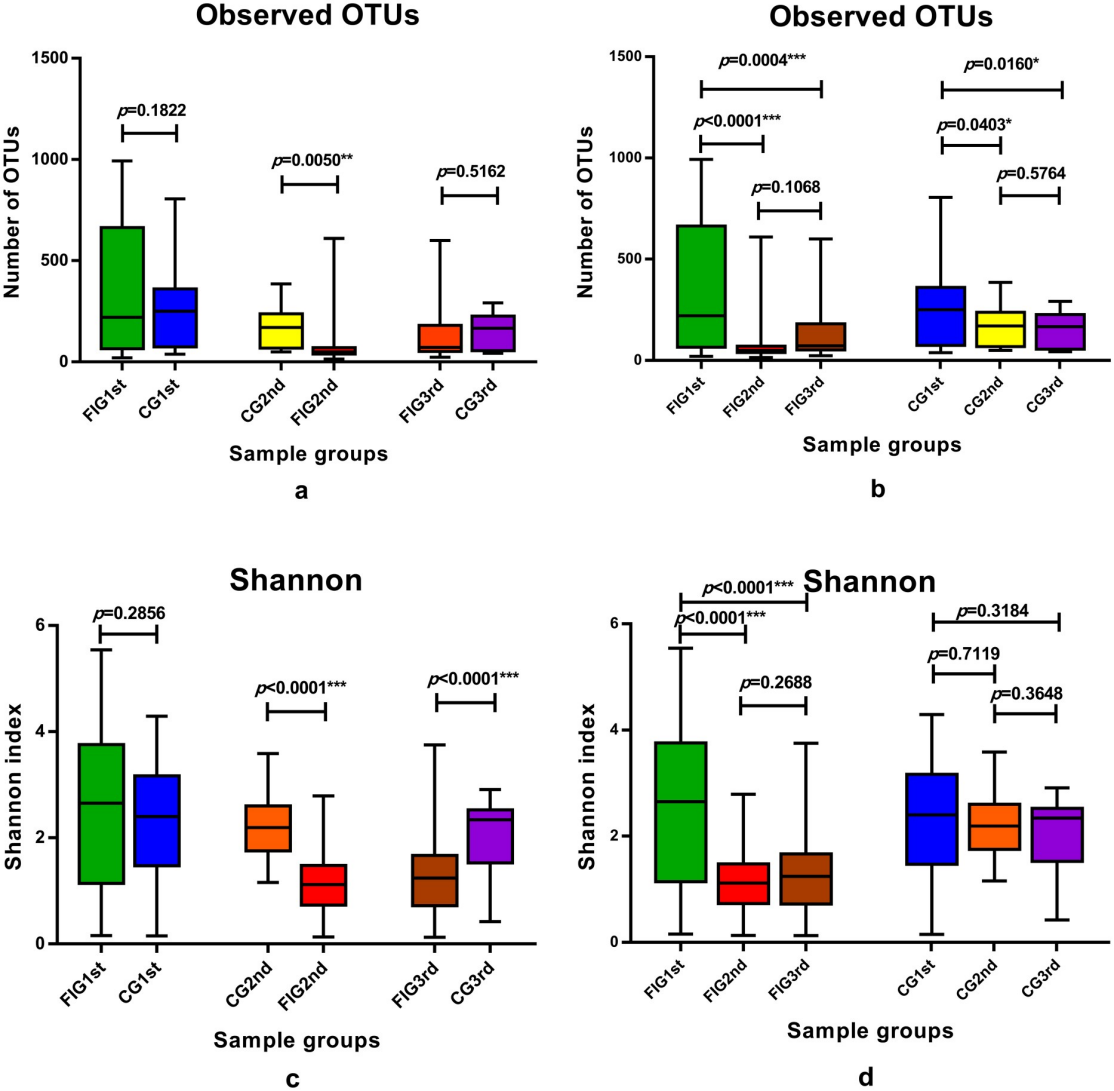
Alpha diversity in the sample groups. The observed OTUs (**1a**,**1b**) and the Shannon index (**1c**, **1d**) in the sample groups of CG1st, CG2nd, CG3rd, FIG1st, FIG2nd and FIG3rd were displayed in different colors. T test was used for differences in pairwise comparison between groups, *p*<0.05 was considered statistically significant.

**Fig 2:**
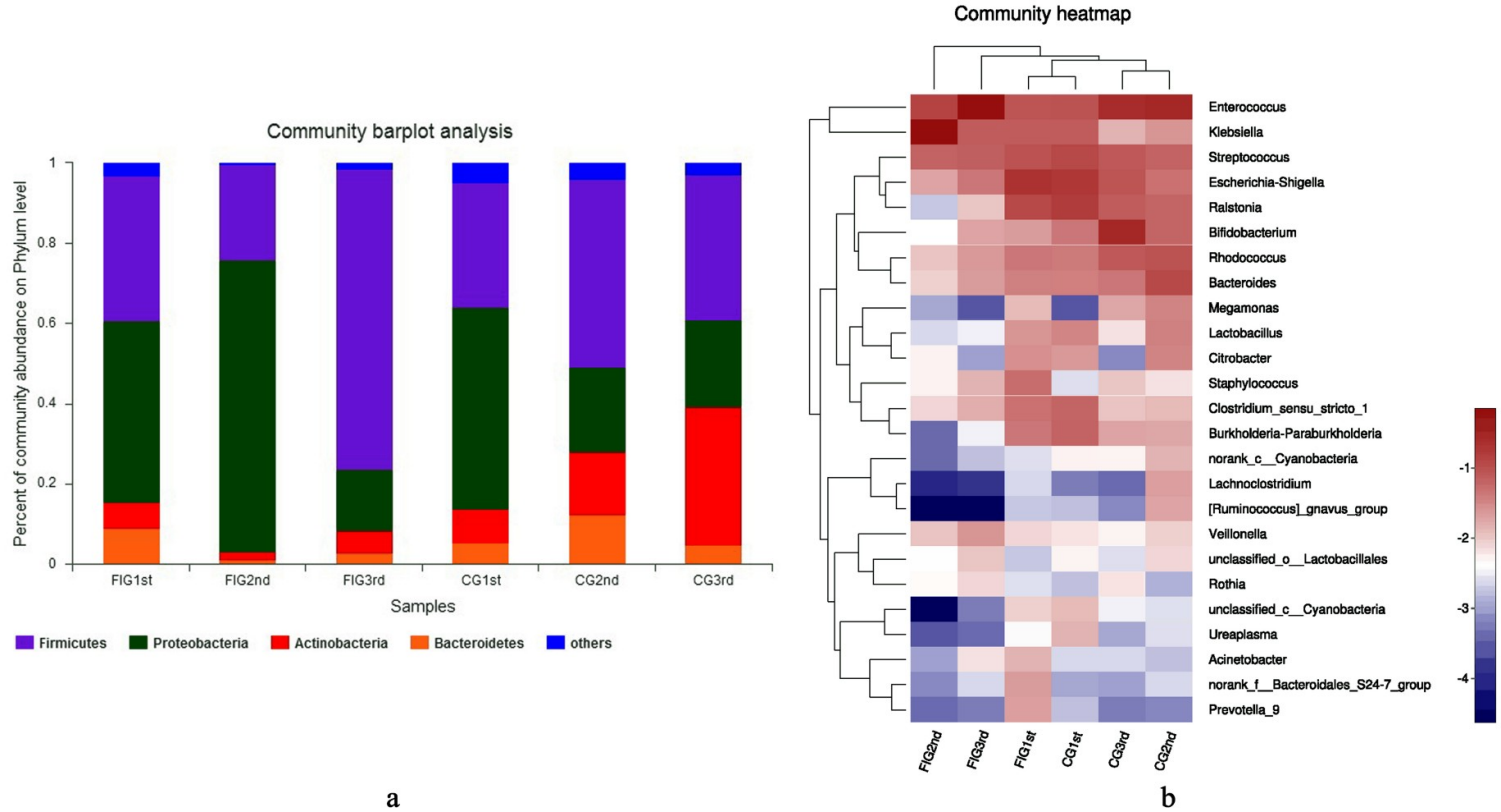
The distribution of microbial groups in the sample groups. The top 4 phyla in the sample groups on the phylum level were displayed in different colors (**2a**). T test was used for differences, *p*<0.05 was considered statistically significant. Heat map of relative abundance of microbes on the genus level in FIG1st, FIG2nd, FIG3rd, CG1st, CG2nd, CG3rd was displayed (**2b**). The warm color represented the microbes with high relative abundance in sample groups while the cold color represented the microbes with low relative abundance.

**Fig 3:**
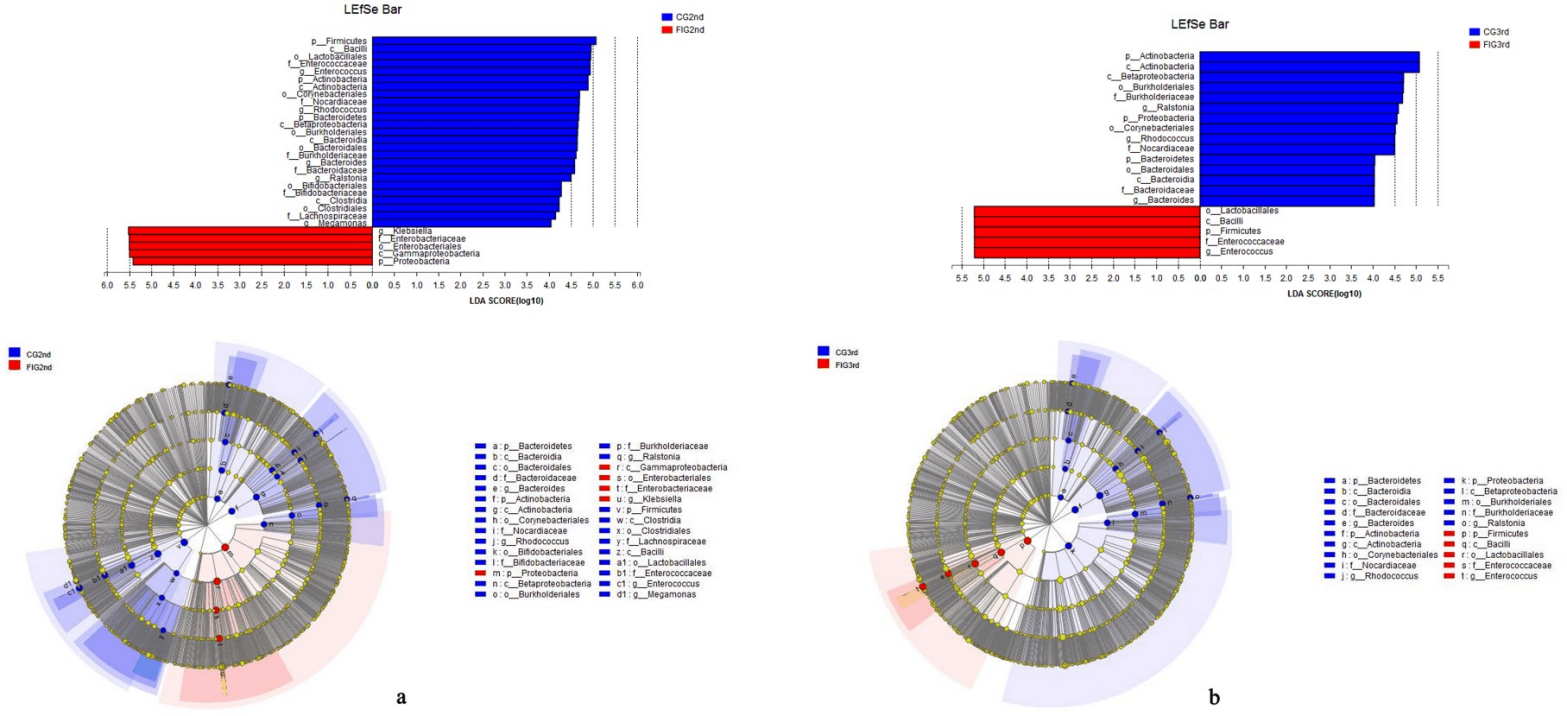
The differential microbes in the samples in the development and prevalence of feeding intolerance. LEfSe analysis by non-parametric factorial Kruskal-Wallis (KW) sum-rank test was used to distinguish FIG2nd and CG2nd (**3a**), FIG3rd and CG3rd (**3b**). The microbes with the LDA score higher than 4 were displayed from the phylum to the genus level.

Fresh meconium samples were collected from the preterm infants in the FIG and the CG groups within 48 hours after birth and classified as FIG1st and CG1st, respectively. FIG1st and CG1st are the samples of the first round of sampling. Fresh fecal samples from preterm infants in the FIG were collected within 24 hours of feeding intolerance being diagnosed and were classified as FIG2nd. We also collected fresh fecal samples from infants of the CG at the same time that feeding intolerance was diagnosed in infants of the FIG and classified these samples as CG2nd. FIG2nd and CG2nd are the samples of the second round of sampling. Fecal samples of infants in the FIG were also collected on the 7th day after feeding intolerance was cured and classified as FIG3rd. We also collected fresh fecal samples from the infants in the CG at the same time of collection as the FIG3rd samples and these samples were classified as CG3rd. A total of 210 fecal samples (41 meconium samples in FIG1st, 29 meconium samples in CG1st, 41 fecal samples in FIG2nd, 29 fecal samples in CG2nd, 41 fecal samples in FIG3rd and 29 fecal samples in CG3rd) collected using disposable sterile swabs from the diapers of preterm infants were then transferred into sterile tubes and stored at -80°C for subsequent DNA extraction.

### Clinical treatments and demographic analysis

The preterm infants in this study were observed for feeding intolerance and other clinical representations during hospitalization. Infants from Xuzhou Maternity and Child Health Care Hospital in the FIG group were supplied with a combined therapy of probiotics and oral erythromycin and the infants from Nanjing Maternity and Child Health Care Hospital in the FIG group were supplied with probiotics as ordered by the physician for the treatment of feeding intolerance. The probiotics we used in treatment included *Bifidobacterium longum*, *Lactobacillus acidophilus*, *Enterococcus faecalis* and *Bacillus subtilis*. The specific dose of probiotics and erythromycin was based on the body weight of each preterm infants. Three antibiotics, sulbencillin-sodium, ceftazidime and erythromycin were used to cure skin infections and urinary infections of the preterm infants during hospitalization. The infants from the FIG continued to be observed for 7 days after feeding intolerance was cured, in case of a relapse. We ensured that all clinical treatments performed on preterm infants at hospital were based on relevant guidelines and regulations of Good Clinical Practice (GCP).

### DNA extraction, PCR amplification and high-throughput sequencing

The bacterial genomic DNA was extracted from samples using a E.Z.N.A. Soil DNA Kit (Omega Bio-tek, Norcross, GA, U.S.), according to the manufacturer’s protocols. Final DNA concentration and purification were determined using a NanoDrop 2000 UV-vis spectrophotometer (Thermo Scientific, Wilmington, USA), and DNA quality was checked using 1% agarose gel electrophoresis. The bacterial primer set of forward primer 338F (5’-ACTCCTACGGGAGGCAGCAG-3’) and reverse primer 806R (5’-GGACTACHVGGGTWTCTAAT-3’) were used to amplify DNA fragments of the 16S rRNA genes. TransStart Fastpfu DNA Polymerase and thermocycler PCR systems (GeneAmp 9700, ABI, USA) were available for PCR. PCR was performed in a 20 μL triplicate mixture containing 4 μL of 5 × FastPfu Buffer, 2 μL of 2.5 mM dNTPs, 0.8 μL of each primer (5 μM), 0.4 μL of FastPfu Polymerase and 10 ng of template DNA. Amplification conditions were as follows: an initial denaturation step at 95°C for 3 minutes, followed by 27 cycles of denaturation at 95°C for 30 seconds, annulation at 55°C for 30 seconds, elongation at 72°C for 45 seconds with a final extension step at 72°C for 5 minute which was halted at 10°C. The resulting PCR products were extracted from a 2% agarose gel and further purified using a AxyPrep DNA Gel Extraction Kit (Axygen Biosciences, Union City, CA, USA) and were quantified using a QuantiFluor-ST Real-time PCR System (Promega, USA) according to the manufacturer’s protocol, before sequencing. 16S rRNA gene sequencing was performed on an Illumina Miseq 300 Platform (Illumina, San Diego, USA) according to standard protocols by Majorbio Bio-pharm Technology Co., Ltd., Shanghai, China. The raw reads were deposited into the NCBI Sequence Read Archive (SRA) database (Accession Number: PRJNA490881 and PRJNA493729). Bacterial DNA and PCR products of all meconium and fecal samples were tested at the same time to avoid multiple freezing.

### Data analysis

All clinical data of the preterm infants were analyzed using SPSS 21.0 software and t test was used to test the significance of difference in pairwise comparisons of the samples. The statistical methods used in the analysis were noted on the annotations of tables or on the legends of figures. Raw fastq files were quality-filtered using Trimmomatic and merged using FLASH under the following criteria: (1) The reads were truncated at any site receiving an average quality score of <20 over a 50bp sliding window. (2) Sequences that overlapped for longer than 10bp were merged according to their overlap with a mismatch of no more than 2bp. (3) Sequences of each sample were separated according to barcodes (exactly matching) and Primers (allowing 2 nucleotide mismatches), and reads containing ambiguous bases were removed. Operational taxonomic units (OTUs) were clustered with a 97% similarity cutoff using UPARSE (version 7.1 http://drive5.com/uparse/) with a novel “greedy” algorithm that simultaneously performs chimera filtering and OTU clustering. The taxonomy of each 16S rRNA gene sequence was analyzed using a RDP Classifier algorithm (http://rdp.cme.msu.edu/) against the Silva (SSU128) 16S rRNA database using a confidence threshold of 70%. All of our laboratory protocols were deposited at protocols.io (https://www.protocols.io/) with its own identifier (DOI): http://dx.doi.org/10.17504/protocols.io.tn4emgw. The supporting information in this work was assigned in DRYAD (https://datadryad.org/) with the 10.5061/dryad.rr18v66.

The histograms, box plots, scatter diagram, heat maps, ROC curves, circos plots with bio-informatic analysis were performed using the free online Majorbio I-Sanger Cloud Platform (www.i-sanger.com) of Shanghai Majorbio Bio-pharm Technology Co., Ltd and GraphPad Prism 7.0. The distance algorithm of Bray-Curtis was used in the analysis of the scatter diagram. Spearman correlation analysis was used in the correlation analysis of the abundance of KEGG pathways and the relative abundance of microbial groups. Linear discriminate analysis (LDA) effect size (LEfSe) by non-parametric factorial Kruskal-Wallis (KW) sum-rank test was based on LEfSe software. 16S rRNA gene prediction was performed based on Phylogenetic investigation of communities by reconstruction of unobserved states (PICRUSt).

## Results and discussions

The demographic information of the preterm infants in the FIG and the CG is shown in Tables 1 and 2. The age of the infants in the FIG was not significantly different from that of the CG at the first (p = 0.2893), second (p = 0.8650) or third (p = 0.1118) rounds of sampling (Table 1). The demographic data of birth weight, gestational age at birth, gender, route of delivery, total antibiotic exposure, dose of probiotics, feedings and development and prevalence of feeding intolerance in preterm infants were recorded from time of birth to the time of the third sampling (Table 2). Birth weight (p = 0.1179) and gestational age at birth (p = 0.4086) of preterm infants in the FIG were not significantly different from that of the CG infants. The gender ratio in the FIG was 19/22 and 14/15 in the CG (Male/Female, M/F) (p = 0.8754). The ratio of the route of delivery was 21/20 in the FIG and 16/13 in the CG (C-section/vaginal) (p = 0.7485). The median exposure time to sulbencillin sodium and ceftazidime was 11 days and 6 days (IQR), respectively in the FIG and 8 days and 9 days (IQR), respectively in the CG. The exposure time to sulbencillin sodium (p = 0.1145) and ceftazidime (p = 0.1011) by the preterm infants in the FIG were not significantly different from that of the CG. The exposure time to erythromycin before the second round of sampling in the FIG was not significantly different from that of the CG (p = 0.3061) while the total exposure time to erythromycin in the FIG was significantly higher than that of the CG (p = 0.0047). The median dose of probiotics for the treatment of feeding intolerance in the FIG was 16 days (IQR). Maternal human milk, formula and parenteral feeding were available for feeding of preterm infants. The median number of feeding days with maternal human milk, formula and parenteral feeding was 11 days, 13 days and 30 days, respectively in the FIG and 14 days, 17 days and 21 days, respectively in the CG. There was no significant difference in the number of feeding days with maternal human milk (p = 0.1029) and formula (p = 0.0959) between the FIG and the CG, but the number of parenteral feeding days of preterm infants in the FIG was significantly higher than that of the CG (p<0.0001).

**Table 1:** The age of the preterm infants at sampling.

	FIG (n = 41)	CG (n = 29)	*p*-value^†^
	Age of infants at sampling,days,median(IQR)		
Age at the first sampling	**FIG1st**	**CG1st**	0.2893
	1(0–2)	1(0–2)	
Age at the second sampling	**FIG2nd**	**CG2nd**	0.8650
	13(7–18)	13(7–19)	
Age at the third sampling	**FIG3rd**	**CG3rd**	0.1118
	50(22–63)	45(23–58)	

T test was used for differences in pairwise comparison between groups, *p*<0.05 was considered statistically significant,

^†^ represented the *p*-value of difference test between CG and FIG.

**Table 2:** Total clinical parameters of preterm infant cohorts admitted in this study.

	FIG (n = 41)	CG (n = 29)	*p*-value^†^
**Birth weight, g, median (IQR)**	1640(900–1900)	1600(1150–1850)	0.1179
**Gestational age at birth, weeks, median (IQR)**	31(26–33)	31(28–33)	0.4086
**Gender, M/F**	19/22	14/15	0.8754
**Route of delivery, C-section/vaginal**	21/20	16/13	0.7485
**Total antibiotic exposure,days,median(IQR),n**			
sulbencillin sodium	11(1–25),25	8(1–20),19	0.1145
ceftazidime	6(2–20),20	9(2–18),17	0.1011
erythromycin (before the second sampling)	5(3–7),9	4(1–7),9	0.3061
erythromycin(total)	14(6–22),27	11(4–18),15	0.0047**
**Dose of probiotics,days,median(IQR),n**	16(8–27),41	-	-
**Feedings, days, median (IQR), n**			
Maternal human milk	11(1–42),41	14(5–37),29	0.1029
Formula	13(5–34),41	17(5–31),29	0.0959
Parenteral feeding	30(16–40),41	21(18–27),29	<0.0001***
**During time of feeding intolerance, days,median(IQR)**	33(2–38)	-	-

T test was used for differences in pairwise comparison between groups, *p*<0.05 was considered statistically significant,

^**†**^ represented the *p*-value of difference test between CG and FIG,

*p<0.05,

**p<0.01,

***p<0.0001.

We analyzed taxonomic alignment results of the OTUs and compared the characteristics of gut microbial community composition of preterm infants in the FIG and the CG. The observed OTUs were compared pairwise between the sample groups of CG1st, FIG1st, CG2nd, FIG2nd, CG3rd and FIG3rd. The number of OTUs in CG1st and CG3rd were not significantly different from that of FIG1st (p = 0.1822) and FIG3rd (p = 0.5162), respectively (Fig 1a). However, the number of OTUs in CG2nd was significantly higher than that of FIG2nd (p = 0.0050). The number of OTUs significantly decreased (compared between FIG1st and FIG2nd, p<0.0001) when feeding intolerance was diagnosed and was not significantly altered after feeding intolerance was cured (compared between FIG2nd and FIG3rd, p = 0.1068) in the samples of preterm infants in the FIG (Fig 1b). In the samples of preterm infants in the CG, the number of OTUs significantly decreased (compared between CG2nd and CG1st, p = 0.0403) and there was no significant difference in the number of OTUs between CG2nd and CG3rd (p = 0.5764). The Shannon index of CG2nd and CG3rd was significantly higher than that of FIG2nd (p<0.0001) and FIG3rd (p<0.0001), respectively (Fig 1c). The Shannon index of CG1st was not significantly different from FIG1st (p = 0.2856). During the development and prevalence of feeding intolerance, the Shannon index of the samples of preterm infants of the FIG significantly decreased when feeding intolerance was diagnosed (compared between FIG1st and FIG2nd, p<0.0001) and had no significant variation after feeding intolerance was cured (compared between FIG2nd and FIG3rd, p = 0.2688) (Fig 1d). In the samples of preterm infants of the CG, the Shannon index had no significant variation (compared between CG2nd and CG1st, p = 0.7119, compared between CG2nd and CG3rd, p = 0.3648).

Microbial groups with a relative abundance higher than 5% in CG1st, FIG1st, CG2nd, FIG2nd, CG3rd and FIG3rd samples at phylum level were Proteobacteria, Firmicutes, Actinobacteria and Bacteroidetes phylum level (Fig 2a). During the development and prevalence of feeding intolerance, Proteobacteria was dominant in FIG1st after birth and in FIG2nd when feeding intolerance was diagnosed. However, after feeding intolerance was cured, Firmicutes was dominant instead of Proteobacteria in FIG3rd. In the samples of preterm infants of the CG, Proteobacteria was dominant in CG1st after birth. Firmicutes was dominant in CG2nd. Firmicutes and Actinobacteria were dominant in CG3rd. The top 25 microbial groups in the samples of CG1st, FIG1st, CG2nd, FIG2nd, CG3rd and FIG3rd at genus level are shown in Fig 2b. In the development and prevelance of feeding intolerance, in FIG1st *Escherichia-Shigella* and *Ralstonia* were dominant, in FIG2nd *Klebsiella* was dominant, while in FIG3rd *Enterococcus* was dominant. In the samples of preterm infants of the CG, in CG1st *Escherichia-Shigella* and *Ralstonia* were dominant, in CG2nd *Enterococcus* was dominant, while in CG3rd *Bifidobacterium* and *Enterococcus* were dominant.

We used the LDA effect size (LEfSe) obtained through a non-parametric factorial Kruskal-Wallis (KW) sum-rank test to further describe the variation among microbial groups during the development and prevalence of feeding intolerance, and predict microbial groups that were potential bio-markers of feeding intolerance in preterm infants. LEfSe can reflect the actual variation of microbial groups between samples better than a t-test. The abundance of only *Escherichia-Shigella* (LDA score higher than 4) in the samples of preterm infants of CG1st was significantly higher than that of FIG1st samples after birth. There were 30 microbial groups (LDA score higher than 4) from phylum level to genus level in the samples of preterm infants of FIG2nd that were significantly different from those of CG2nd when feeding intolerance was diagnosed (Fig 3). After feeding intolerance was cured, 20 microbial groups of FIG3rd were significantly discriminated from those of CG3rd. The relative abundance of many microbial groups both in the CG and the FIG (LDA score higher than 4) had been significantly and drastically altered when feeding intolerance was diagnosed (S1 Fig). After feeding intolerance was cured, unlike the significant variation in many microbial groups between FIG2nd and FIG3rd, only the relative abundance of Actinobacteria of CG3rd was significantly different from CG2nd, indicating that the community composition of gut microbiota of preterm infants of the CG was relatively stable at this stage (Figures B and C in S1 Fig). The relative abundance of *Klebsiella* in the FIG, significantly increased when feeding intolerance was diagnosed and significantly decreased after feeding intolerance was cured. In the CG, the relative abundance of *Klebsiella* significantly decreased and was maintained at a very low relative abundance in CG3rd. There was also no significant difference in the relative abundance of *Klebsiella* between CG1st and FIG1st after birth but the relative abundance of *Klebsiella* in FIG2nd was significantly higher than that of CG2nd. Therefore, in this study the proliferation of *Klebsiella* was possibly related to the occurrence of feeding intolerance in the preterm infants of the FIG.

**Fig 4:**
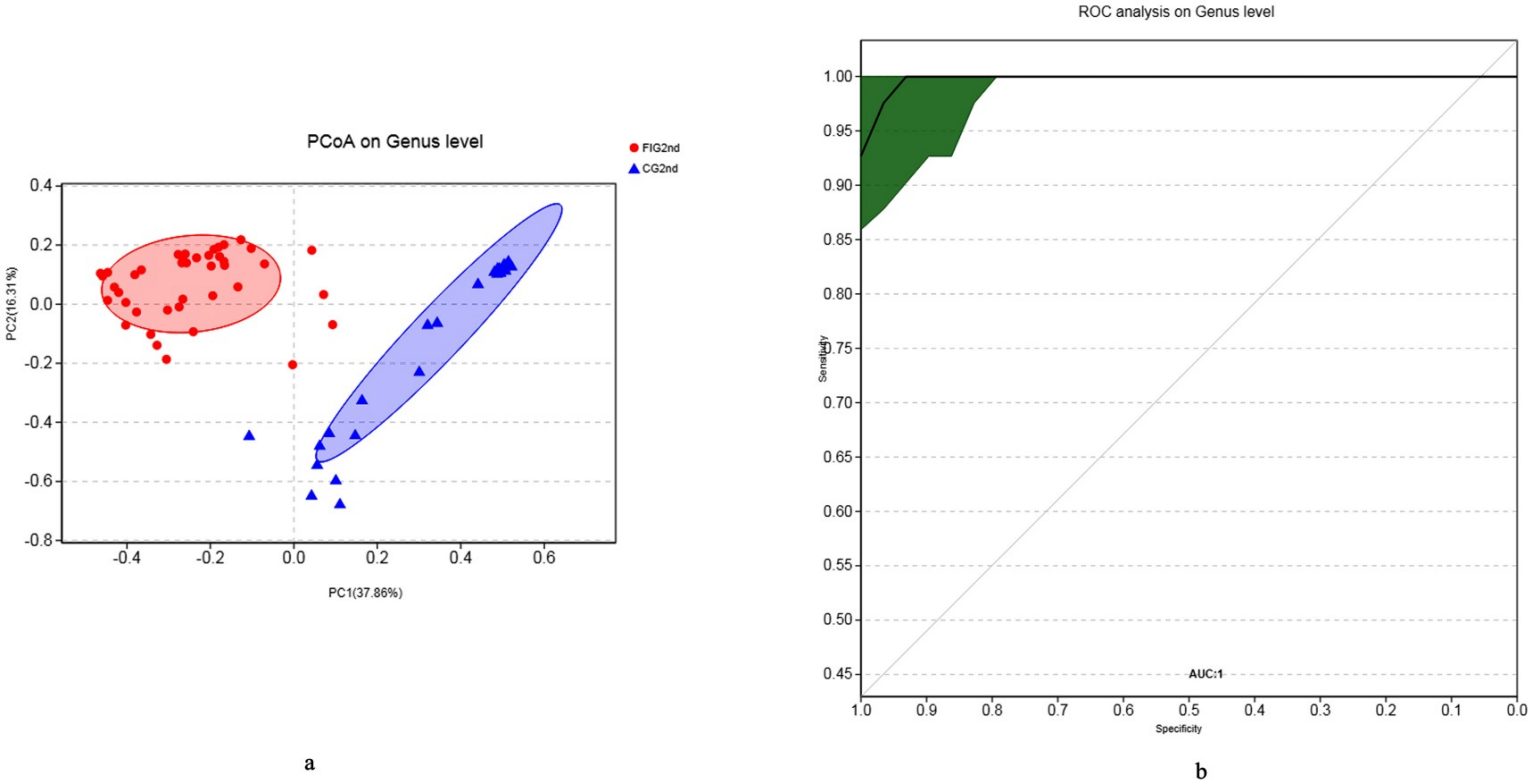
The PCoA between sample groups and ROC analysis of *Klebsiella* when FI was diagnosed. The PCoA on the genus level between FIG2nd and CG2nd was displayed (**4a**). The ROC of *Klebsiella* in FIG2nd and CG2nd was displayed (**4b**). The x axis represented the specificity and the y axis represented the sensibility. The green area was represented the 95% confidence interval (CI).

In order to find potential microbial biomarkers at different phases of the development and prevalence of feeding intolerance, we evaluated the sensitivity and specificity of potential bio-markers using random forest analysis and a receiver operating characteristic curve (ROC). After birth, *Collinsella* (with the highest variable importance in CG1st and FIG1st: 4.124, AUC = 0.68), a genus belonging to Actinobacteria, was more effective in discriminating between CG1st and FIG1st than *Escherichia-Shigella* (variable importance: 1.303, AUC = 0.64) (Figures A and B in S2 Fig). However, the meconium samples of FIG1st and CG1st, collected from preterm infants within 48 hours of birth from different hospitals were more likely to be influenced by the hospital environment, and accordingly the microbial groups of the meconium samples seemed to be significantly hospital-specific (Figures A and B in S3 Fig). Moreover, *Collinsella* was effective as a microbe to distinguish between FIG1st and CG1st, in the samples from Xuzhou Maternity and Child Health Care Hospital (AUC = 0.81) but useless in the samples from Nanjing Maternity and Child Health Care Hospital (AUC = 0.45), indicating that it seemed difficult to find a consistent microbial bio-marker at different sites for the prediction of feeding intolerance after birth. Therefore, for the sample size in this study, the hospital-specific meconium samples were inadequate to enable *Collinsella* (maintaining a very low relative abundance in all samples) to be a potential biomarker to distinguish FIG1st from CG1st after birth. When feeding intolerance was diagnosed, CG2nd was easily distinguished from FIG2nd because the relative abundances of *Klebsiella* were effective in this regard (AUC = 1) (Fig 4a and 4b). Furthermore, samples from different hospitals in FIG2nd showed a high degree of similarity (Figure A in S4 Fig). The relative abundance of *Klebsiella* was also effective in distinguishing FIG2nd from CG2nd at Xuzhou Maternity and Child Health Care Hospital (AUC = 1) and at Nanjing Maternity and Child Health Care Hospital (AUC = 1). It was effective even when comparing FIG2nd_X with CG2nd_N (AUC = 1), and FIG2nd_N with CG2nd_X (AUC = 0.98) (Figures B and C in S4 Fig). This suggests that in this study *Klebsiella* was a potential biomarker when feeding intolerance was diagnosed in preterm infants.

**Fig 5:**
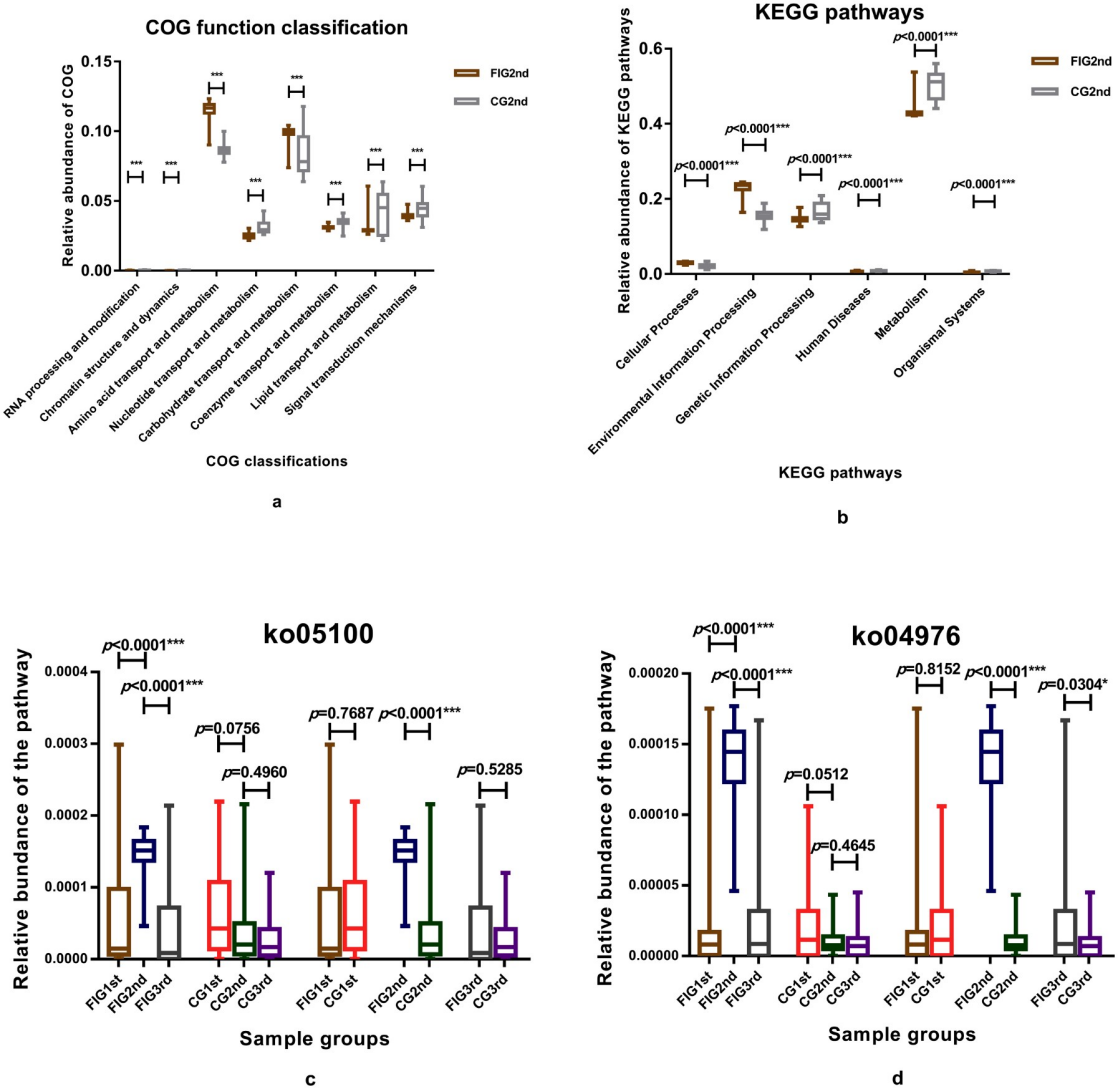
Relative abundance of the COG classifications and KEGG pathways in sample groups. T test was used for differences, *p*<0.05 was considered statistically significant. The COG classifications with significant differences (**5a**) and the KEGG pathways in sample groups when feeding intolerance was diagnosed were displayed (**5b**). Relative abundance of ko05100 and ko04976 in sample groups were compared and represented in different colors (**5c**, **5d**).

PICRUSt was used to predict bacterial functions in the samples from preterm infants of the CG and the FIG. When feeding intolerance was diagnosed, the relative abundance of COG function classifications were compared and the COG function classifications with significant differences in the relative abundances between CG2nd andFIG2nd are shown in Fig 5a. The relative abundances of COG function classifications of RNA processing and modification, chromatin structure and dynamics, nucleotide transport and metabolism, coenzyme transport and metabolism, lipid transport and metabolism, signal transduction mechanisms of CG2nd were significantly higher than that of FIG2nd (p<0.0001). However, the relative abundances of amino acid transport and metabolism, carbohydrate transport and metabolism of FIG2nd were significantly higher than those of CG2nd (p<0.0001). We also compared the difference in the relative abundance of KEGG pathways of samples between CG2nd and FIG2nd (Fig 5b). The relative abundances of KEGG pathways of cellular processes and environmental information processing of FIG2nd were significantly higher than that of CG2nd (p<0.0001) while the relative abundance of KEGG pathways of genetic information processing, human diseases, metabolism and organismal systems of CG2nd were significantly higher than those of FIG2nd (p<0.0001). Among them, during the development and prevalence of feeding intolerance, the relative abundances of KEGG pathways of bacterial invasion of epithelial cells (ko05100) and bile secretion (ko04976) significantly increased when feeding intolerance was diagnosed (p<0.0001) and significantly decreased after feeding intolerance was cured (p<0.0001) (Fig 5c and 5d). There was no significant variation between the relative abundances of ko05100 in the samples of preterm infants from the CG of this study (compared between CG1st and CG2nd, p = 0.0756; compared between CG2nd and CG3rd, p = 0.4960). Additionally, the relative abundance of ko05100 was not significantly different between CG1st and FIG1st (p = 0.7687). When feeding intolerance was diagnosed, the relative abundance of ko05100 of FIG2nd was significantly higher than that of CG2nd (p<0.0001), while the relative abundance of ko05100 of FIG3rd was not significantly different from that of CG3rd after feeding intolerance was cured (p = 0.5285). The relative abundance of ko04976 of CG2nd was not significantly different from CG1st (p = 0.0512). The relative abundance of ko04976 was also not significantly different between CG1st and FIG1st after birth (p = 0.8152). However, the relative abundance of ko04976 of FIG2nd and FIG3rd were significantly higher than that of CG2nd (p<0.0001) and CG3rd (p = 0.0304), respectively. Notably, when feeding intolerance was diagnosed, the relative abundance of *Klebsiella* was significantly relevant to the relative abundance of ko05100 (r = 0.963, p<0.0001) and ko04976 (r = 0.999, p<0.0001) in the samples of FIG2nd. *Klebsiella* also contributed to 94.8% of the mean abundance of ko05100 and 99.6% of the mean abundance of ko0496 of FIG2nd, suggesting that *Klebsiella* was significantly relevant to the significant increase in the abundance of ko05100 and ko04976 in the samples of preterm infants of the FIG, when feeding intolerance was diagnosed.

## Conclusion

In this study, we aimed to detect the differences in gut microbiota communities of preterm infants diagnosed with feeding intolerance and preterm infants not diagnosed with feeding intolerance, and attempted to predict biomarkers and the diagnostic markers of feeding intolerance from the microbial groups. Gut microbial communities were dramatically altered when feeding intolerance was diagnosed both in preterm infants of the FIG and the CG, and we demonstrated that *Klebsiella* is relevant to feeding intolerance of preterm infants. The relative abundance of many COG classifications and KEGG pathways of preterm infants diagnosed with feeding intolerance were significantly different from that of preterm infants not diagnosed with feeding intolerance. In this study, KEGG pathways of bacterial invasion of epithelial cells (ko05100) and bile secretion (ko04976) were significantly relevant to *Klebsiella*.

The dysbiotic expansion of Proteobacteria is a potential diagnostic biomarker of epithelial dysfunction in human gut [10,25]. In our study, the alpha diversity, including observed number of OTUs and Shannon index significantly decreased when feeding intolerance was diagnosed. The significant increase in the relative abundance of KEGG pathways of bacterial invasion of epithelial cells and bile secretion indicate a higher potential risk of damage to intestinal epithelial cells, intestinal dysfunction and an increase in bile secretion, which are related to the appearance of feeding intolerance in the preterm infants [26–30]. Preterm infants with presentations of a gastric residual volume of more than 50% of the previous feeding volume and abdominal distension were diagnosed as feeding intolerant. However, the collection of gastric fluid to weigh gastric residual volume can probably cause pain to preterm infants and may result in misdiagnosis of feeding intolerance. Therefore, apart from improving the diagnostic criteria of feeding intolerance, a biomarker is also necessary to distinguish between infants suffering from feeding intolerance and healthy infants. The relative abundance of the genus *Klebsiella* which belongs to Proteobacteria was effective in distinguishing preterm infants with feeding intolerance from healthy infants when feeding intolerance appeared, suggesting that according to the results of our study, *Klebsiella* is a potential diagnostic marker of feeding intolerance in preterm infants. *Klebsiella* is an opportunistic pathogen of humans, and the intestinal environment of preterm infants after birth is not strictly anaerobic, often leading to an increase of Proteobacteria in the human gut [25].

Obligate anaerobes (including *Bifidobacterium*, *Bacteroides* and *Lactobacillus*) are thought to be dominant in the gut of healthy infants after birth [16,26]. However in our study, aerobes and facultative anaerobes, including *Klebsiella*, *Ralstonia*, *Rhodococcus*, *Escherichia*-*Shigella* and *Burkholderia*-*Paraburkholderia*, were dominant in the gut microbial communities of all preterm infants after birth, suggesting a potential indication of dysbacteriosis which may cause long-term impacts on microbial evolution in human gut. The relative abundance of *Enterococcus* in healthy preterm infants was significantly higher than that of preterm infants with feeding intolerance when feeding intolerance was diagnosed, suggesting that *Enterococcus* probably plays an important role in maintaining intestinal homeostasis in humans [31]. Moreover, the lower microbial diversity, the lower relative abundance of Firmicutes, and the significantly increased relative abundance of *Klebsiella* in preterm infants when feeding intolerance was diagnosed that we detected in this study is also a distinct feature of preterm infants diagnosed with NEC in previous studies [21,32,33]. This suggests that the characteristics of the gut microbial community composition of preterm infants when feeding intolerance was diagnosed is similar to that of preterm infants with NEC.

Additionally, we were concerned that the community composition of FIG3rd was still very different from that of CG3rd after feeding intolerance was cured. Notably, preterm infants from FIG_X were treated for feeding intolerance with erythromycin and probiotics, while infants from FIG_N were treated for feeding intolerance with only probiotics. Although erythromycin has been demonstrated to be effective in improving feeding intolerance and has been widely used, the possible side-effects caused by erythromycin on the microbial community should be of concern [2,5]. After feeding intolerance was cured, 17 microbial groups (with a LDA score higher than 4) that discriminated between FIG3rd_X and FIG3rd_N were detected (Figure A in S5 Fig). In FIG3rd_N without using erythromycin, the relative abundance of *Bifidobacterium*, which is beneficial to the human gut, was significantly higher than in FIG3rd_X treated with erythromycin. There was however no significant difference in the relative abundance of *Bifidobacterium* between FIG_X and FIG_N after birth or when feeding intolerance was diagnosed (Figures B and C in S5 Fig). Additionally, individual data of infants from FIG_X was not significantly different from infants of FIG_N (S1 and S2 Tables). Erythromycin, as an antibiotic, was likely to be a major potential confounding variable of the gut microbial community composition, which can probably have a long-term effect on gut microbial evolution [34]. Also, according to the available data, only the doses of erythromycin using (days) in FIG_X were significantly different from FIG_N (S1 and S2 Tables). However, there were more potential influence factors which probably impacted the the shift in *Klebsiella* in different hospitals. Besides, although the antibiotic influence of erythromycin in *Klebsiella* should not be ignored, whether the small doses of erythromycin on treating FI in FIG could entirely caused to such a significant shift in *Klebsiella* was still unclear. Therefore, the shift in relative abundance of *Klebsiella* from FIG2nd to FIG3rd was more likely to be both a representation of healing of FI and an effect caused by antibiotic. Further studies are obviously needed to explore the deep relationship between erythromycin use and the shift in *Klebsiella*. To date, the efficacy and the mechanism of action of erythromycin in treating FI infants is still not completely clear. Therefore, erythromycin use in infants should be considered cautiously and more studies are needed on the treatment of FI.

The results of this study can provide a theoretical basis for the prediction, diagnosis and treatment of feeding intolerance from a micro-ecological and precision medicine view. However, the community composition of first bacterial colonization of preterm infants was highly influenced by many factors such as the hospital environment or the population attending the hospital, leading to insignificant and random characteristics of the gut microbial community composition of preterm infants after birth [35–37]. Additionally, The inter-specific interactions among the microbial species should be studied in a further study to resolve complicated inter-specific relationships and dynamics of the microbial ecosystem of the human gut in order to resolve FI, and these results may help us to further understand the interrelationship between the microbial ecosystem and the host. Furthermore, we need to confirm whether *Klebsiella* was consistently dominant in preterm infants not involved in this study when feeding intolerance was diagnosed. Therefore, in future more samples and more studies will be needed to verify this potential diagnostic maker, and to detect other potential biomarkers of feeding intolerance.

## Supporting information

S1 Fig The altered microbes in the sample groups. LEfSe analysis by non-parametric factorial Kruskal-Wallis (KW) sum-rank test was used to distinguish FIG2nd and FIG1st (**a**), FIG2nd and FIG3rd (**b**), CG1st and CG2nd (**c**), CG2nd and CG3rd (**d**). The microbes with the LDA score higher than 4 were displayed from the phylum to the genus level.
(TIF)

S2 Fig The sensitivity and specificity of the potential bio-markers between CG1st and FIG1st. ROC analysis for the sensitivity and specificity of *Escherichia-Shigella* (**a**) and *Collinsella* (**b**) on the genus level between CG1st and FIG1st after birth.
(TIF)

S3 Fig PCoA of the sample groups on the genus level. The scatter plots of PCoA by Bray-Curtis between FIG1st_X and FIG1st_N (**a**), CG1st_X and CG1st_N (**b**) were displayed.
(TIF)

S4 Fig PCoA and ROC analysis in the samples groups when FI was diagnosed. The scatter plots of PCoA by Bray-Curtis in FIG2nd_X, FIG2nd_N, CG2nd_X, CG2nd_N (**a**) was displayed. The AUC of *Klebsiella* between FIG2nd_X and CG2nd_N was 1 (**b**) and 0.98 between FIG2nd_N and CG2nd_X (**c**).
(TIF)

S5 Fig The differential microbes in the samples from different hospitals in the development and prevalence of feeding intolerance. LEfSe analysis by non-parametric factorial Kruskal-Wallis (KW) sum-rank test was used to distinguish FIG3rd_N and FIG3rd_X (**a**), FIG1st_N and FIG1st_X (**b**), FIG2nd_X and FIG2nd_N (**c**). The microbes with the LDA score higher than 4 were displayed from the phylum to the genus level.
(TIF)

S1 Table The age of the preterm infants at sampling. T test was used for differences in pairwise comparison between groups, *p*<0.05 was considered statistically significant, † represented the p-value of difference test between the FIG_X and the FIG_N.
(DOCX)

S2 Table Total clinical parameters of preterm infant cohorts in the FIG_N and the FIG_X. T test was used for differences in pairwise comparison between groups, *p*<0.05 was considered statistically significant, † represented the p-value of difference test between the FIG_X and the FIG_N. ***p<0.0001.
(DOCX)
