# Supplementary figures and images for: Feeding intolerance alters the gut microbiota of preterm infants
Source: PLoS One. 2019 Jan 22;14(1):e0210609. doi: 10.1371/journal.pone.0210609 (PMC6342312; doi:10.1371/journal.pone.0210609)

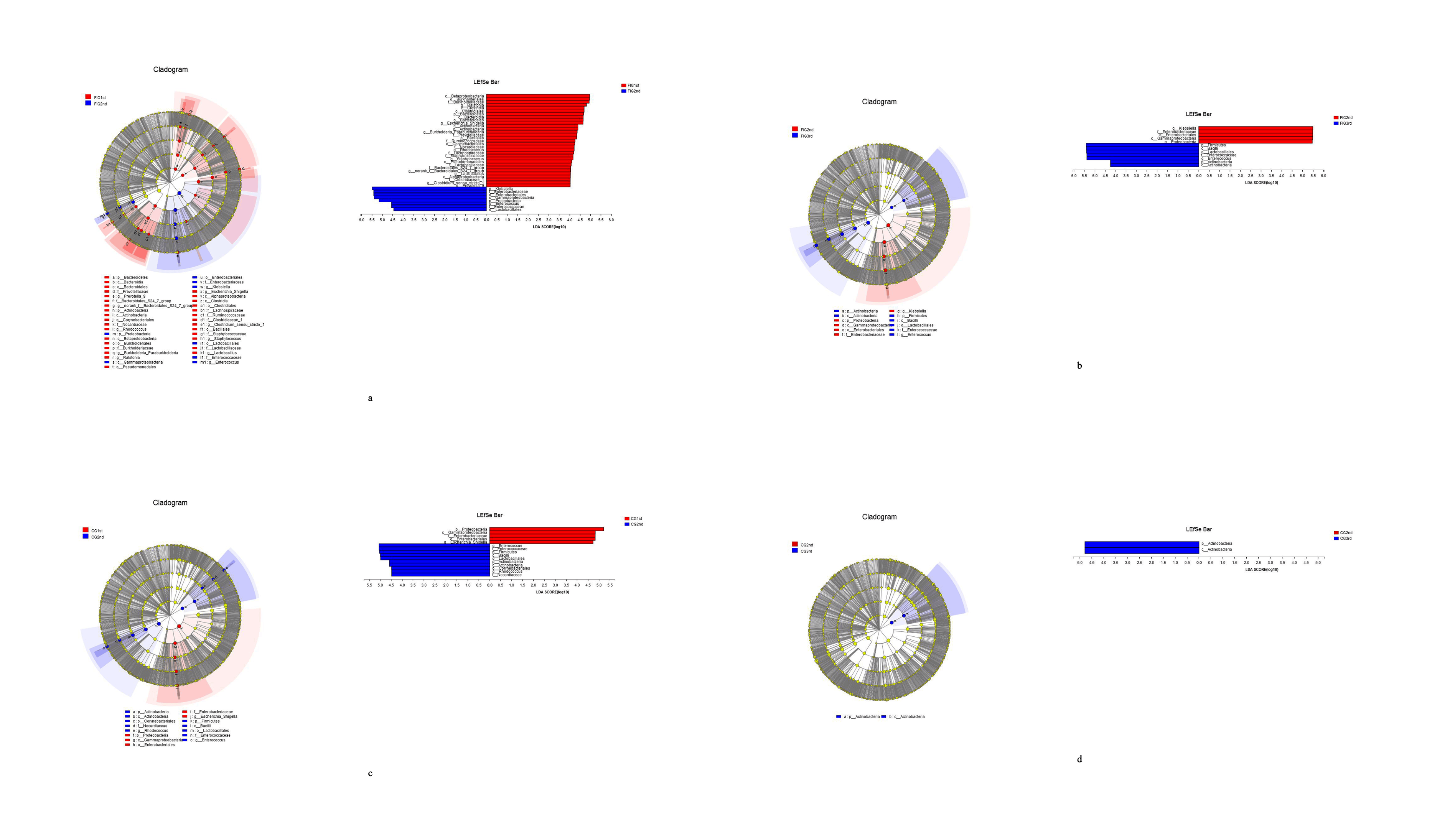

Supplement: S1 Fig — LEfSe analysis by non-parametric factorial Kruskal-Wallis (KW) sum-rank test was used to distinguish FIG2nd and FIG1st (a), FIG2nd and FIG3rd (b), CG1st and CG2nd (c), CG2nd and CG3rd (d). The microbes with the LDA score higher than 4 were displayed from the phylum to the genus level. (TIF) [file pone.0210609.s001.tif]

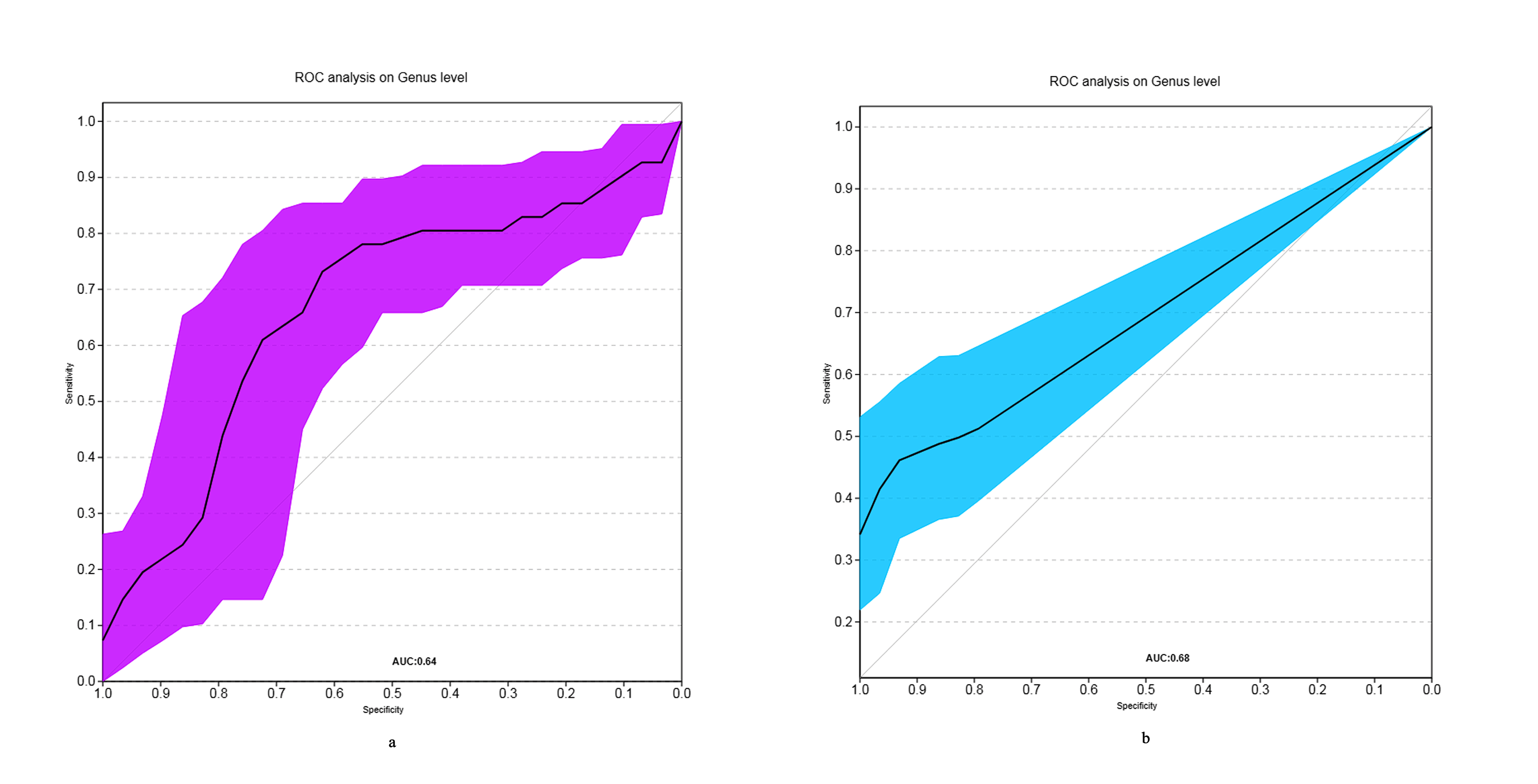

Supplement: S2 Fig — ROC analysis for the sensitivity and specificity of Escherichia-Shigella (a) and Collinsella (b) on the genus level between CG1st and FIG1st after birth. (TIF) [file pone.0210609.s002.tif]

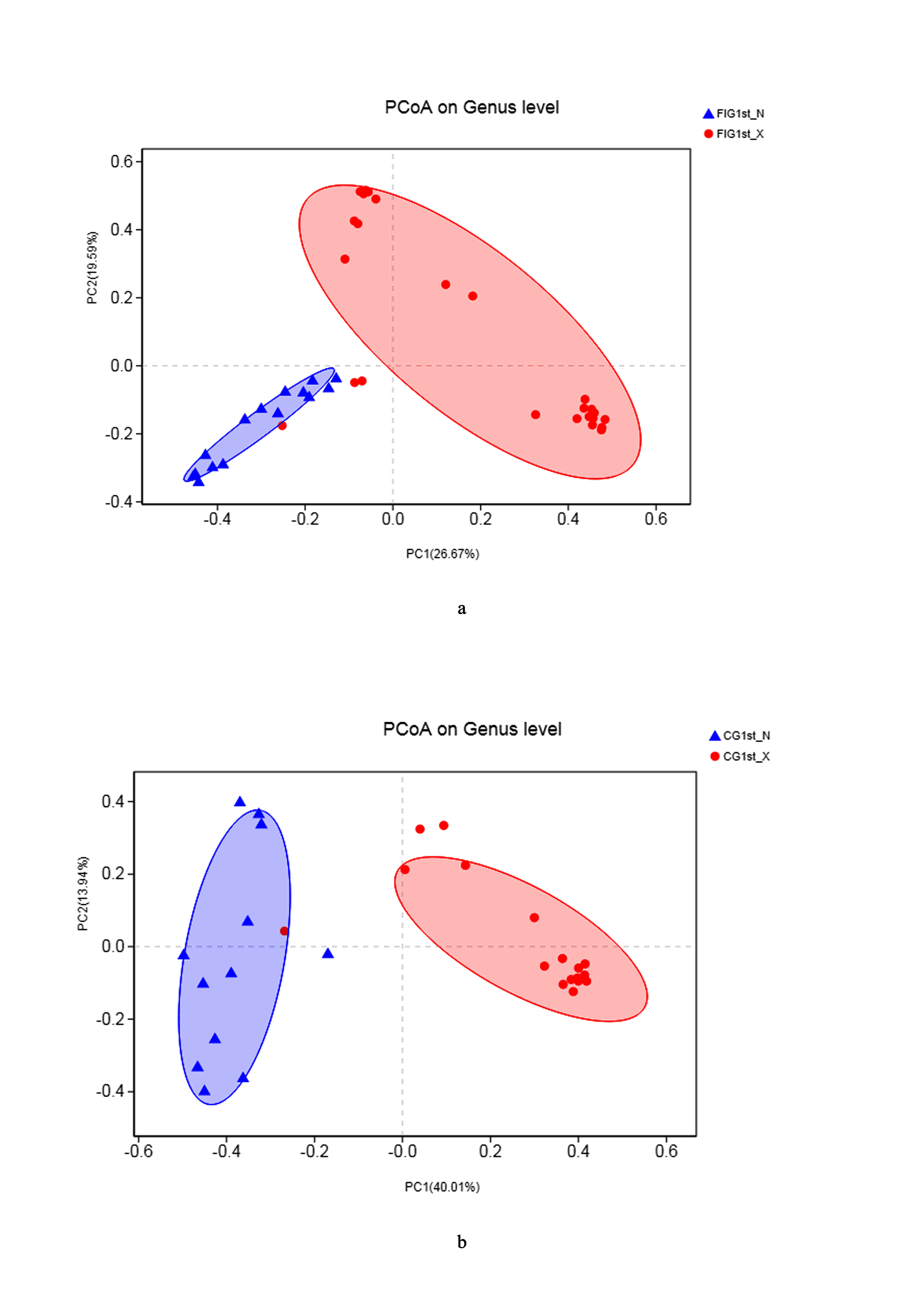

Supplement: S3 Fig — The scatter plots of PCoA by Bray-Curtis between FIG1st_X and FIG1st_N (a), CG1st_X and CG1st_N (b) were displayed. (TIF) [file pone.0210609.s003.tif]

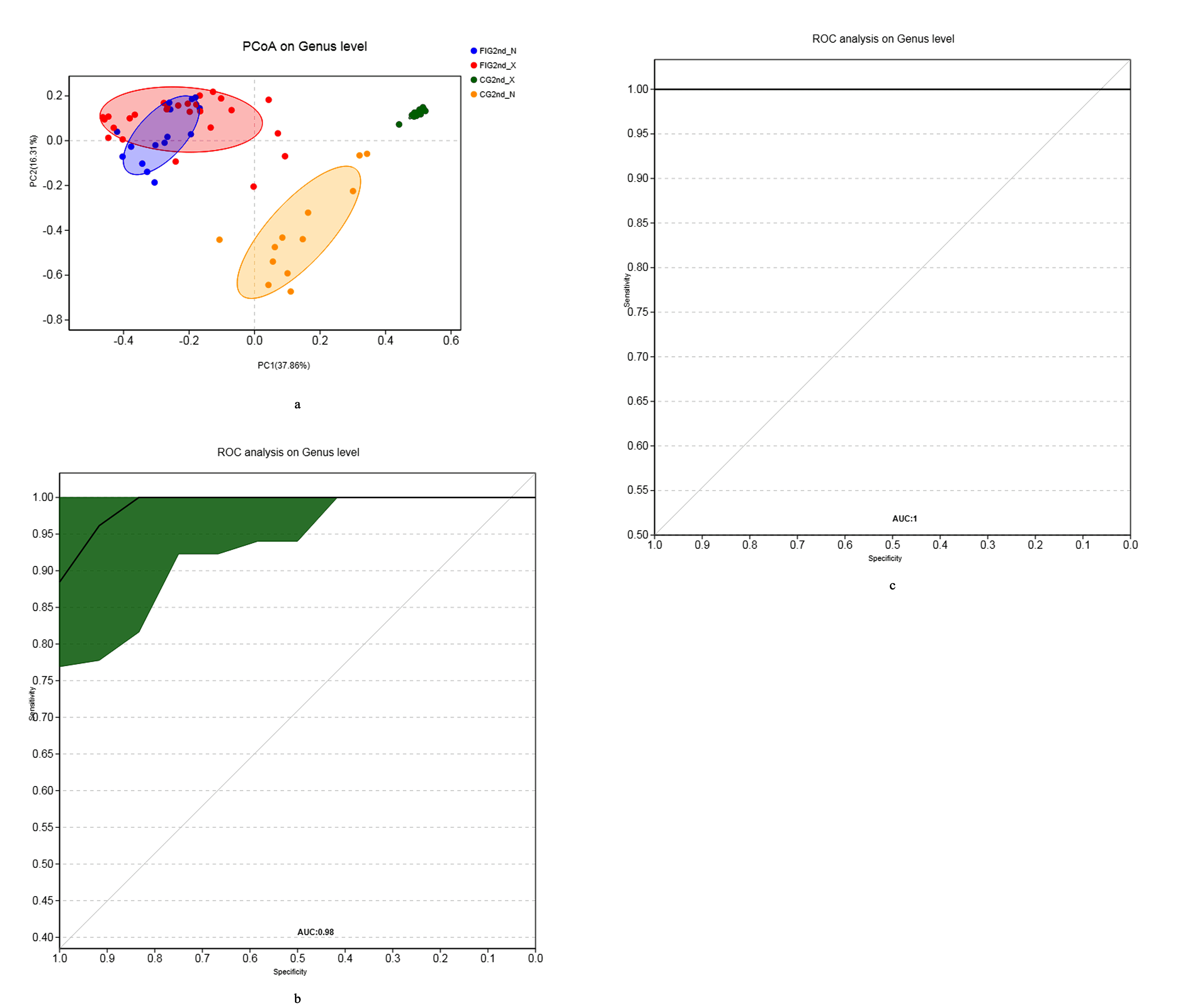

Supplement: S4 Fig — The scatter plots of PCoA by Bray-Curtis in FIG2nd_X, FIG2nd_N, CG2nd_X, CG2nd_N (a) was displayed. The AUC of Klebsiella between FIG2nd_X and CG2nd_N was 1 (b) and 0.98 between FIG2nd_N and CG2nd_X (c). (TIF) [file pone.0210609.s004.tif]

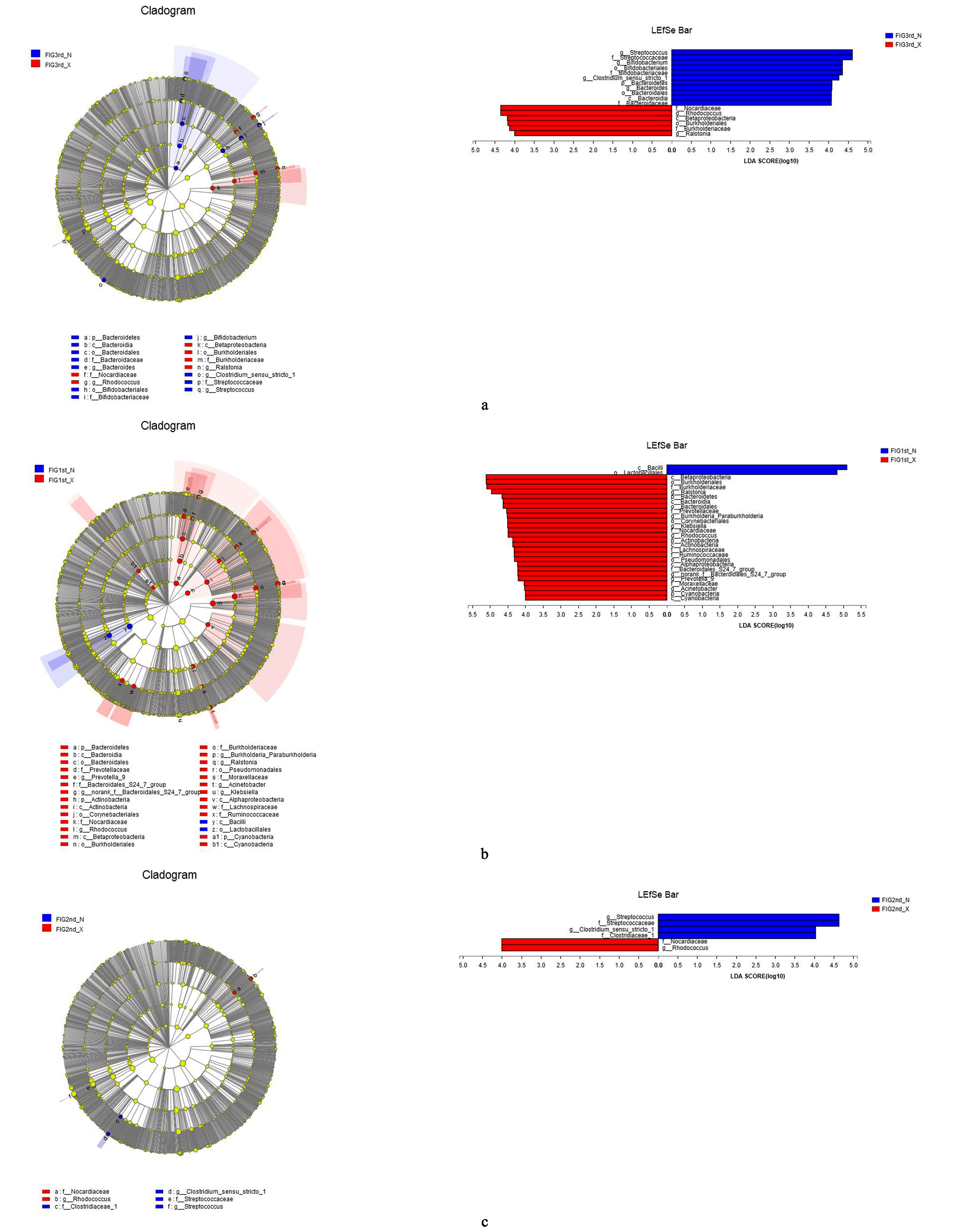

Supplement: S5 Fig — LEfSe analysis by non-parametric factorial Kruskal-Wallis (KW) sum-rank test was used to distinguish FIG3rd_N and FIG3rd_X (a), FIG1st_N and FIG1st_X (b), FIG2nd_X and FIG2nd_N (c). The microbes with the LDA score higher than 4 were displayed from the phylum to the genus level. (TIF) [file pone.0210609.s005.tif]
