# Supplementary material for: Feeding intolerance alters the gut microbiota of preterm infants
Source: PLoS One. 2019 Jan 22;14(1):e0210609. doi: 10.1371/journal.pone.0210609 (PMC6342312; doi:10.1371/journal.pone.0210609)
Supplement: S1 Table — T test was used for differences in pairwise comparison between groups, p<0.05 was considered statistically significant, † represented the p-value of difference test between the FIG_X and the FIG_N. (DOCX) [file pone.0210609.s006.docx]

**S1 Table** **1**. The age of the preterm infants at sampling.

|  | FIG_X (n=26) | FIG_N (n=15) | *p*-value† |
| --- | --- | --- | --- |
|  | Age of infants at sampling,days,median(IQR) | |  |
| Age at the first sampling | FIG1st_X | FIG1st_N | 0.4327 |
|  | 1(0-2) | 1(0-2) |  |
| Age at the second sampling | FIG2nd_X | FIG2nd_N | 0.1676 |
|  | 10(7-18) | 14(8-18) |  |
| Age at the third sampling | FIG3rd_X | FIG3rd_N | 0.5575 |
|  | 50(22-63) | 50(33-60) |  |

T test was used for differences in pairwise comparison between groups, *p*<0.05 was considered statistically significant, † represented the p-value of difference test between the FIG_X and the FIG_N.
