# Supplementary material for: Feeding intolerance alters the gut microbiota of preterm infants
Source: PLoS One. 2019 Jan 22;14(1):e0210609. doi: 10.1371/journal.pone.0210609 (PMC6342312; doi:10.1371/journal.pone.0210609)
Supplement: S2 Table — T test was used for differences in pairwise comparison between groups, p<0.05 was considered statistically significant, † represented the p-value of difference test between the FIG_X and the FIG_N. ***p<0.0001. (DOCX) [file pone.0210609.s007.docx]

**S2 Table.** Total clinical parameters of preterm infant cohorts in the FIG_N and the FIG_X.

|  | FIG_X (n=26) | | FIG_N (n=15) | | *p*-value**^†^** |
| --- | --- | --- | --- | --- | --- |
| **Birth weight, g, median (IQR)** | 1600(900-1900) | | 1650(1510-1820) | | 0.2215 |
| **Gestational age at birth, weeks, median (IQR)** | 31(26-33) | | 31(30-33) | | 0.5074 |
| **Gender, M/F** | 11/15 | | 8/7 | | 0.5075 |
| **Route of delivery, C-section/vaginal** | 14/12 | | 7/8 | | 0.6673 |
| **Total antibiotic exposure,days,median(IQR),n** | | | | | |
| sulbencillin sodium | 11(3-25),20 | | 10(1-23),5 | | 0.4195 |
| ceftazidime | 8(2-20),13 | | 4(2-7),7 | | 0.0664 |
| erythromycin (before the second sampling) | 5(3-7),6 | | 6(4-6),3 | | 0.6491 |
| erythromycin(total) | 14(8-22),26 | | 6(6-6),1 | | <0.0001*** |
| **Dose of probiotics,days,median(IQR),n** | 15(12-22),26 | 16(8-27),15 | | | 0.4845 |
| **Feedings, days, median (IQR), n** | | | | | |
| Maternal human milk | 9(1-29),26 | | 13(7-42),15 | | 0.3255 |
| Formula | 13(5-34),26 | | 12(7-26),15 | | 0.7404 |
| Parenteral feeding | 30(16-38),26 | | | 34(27-40),15 | 0.0674 |
| **During time of feeding intolerance, days,median(IQR)** | 33(2-38),26 | | | 32(18-38),15 | 0.9613 |

T test was used for differences in pairwise comparison between groups, *p*<0.05 was considered statistically significant, † represented the p-value of difference test between the FIG_X and the FIG_N. *** *p*<0.0001.
